# Lack of both androgen receptor and forkhead box A1 (FOXA1) expression is a poor prognostic factor in estrogen receptor-positive breast cancers

**DOI:** 10.18632/oncotarget.20937

**Published:** 2017-09-15

**Authors:** Seho Park, Eunjin Koh, Ja Seung Koo, Seung Il Kim, Byeong-Woo Park, Kyung-Sup Kim

**Affiliations:** ^1^ Department of Surgery, Yonsei University College of Medicine, Seoul, South Korea; ^2^ Frontier Research Institute of Convergence Sports Science, Yonsei University, Seoul, South Korea; ^3^ Department of Biochemistry and Molecular Biology, Institute for Genetic Science, Integrated Genomic Research Center for Metabolic Regulation, Yonsei University College of Medicine, Seoul, South Korea; ^4^ Department of Pathology, Yonsei University College of Medicine, Seoul, South Korea

**Keywords:** androgen receptor, breast neoplasms, estrogen receptor, forkhead box A1, prognosis

## Abstract

The present study aimed to examine the associations between androgen receptor (AR) and forkhead box A1 (FOXA1) and to investigate clinicopathological features and survival according to both biomarker status in estrogen receptor (ER)-positive breast cancers using *in vitro* study, patient cohort data, and the cBioPortal for Cancer Genomics and Kaplan-Meier Plotter websites. Experiments using T47D and ZR75-1 demonstrated AR-overexpressing cell lines decreased in cell proliferation through downregulation of ER, but FOXA1 did not change. Knockdown of FOXA1 resulted in a significantly reduced cell viability. Patients with immunohistochemically AR(-)/FOXA1(-) tumor frequently showed node metastasis, high grade, and high Ki-67 proliferation, therefore, significantly worse survival in ER-positive disease. AR and FOXA1 mRNA levels were significantly higher in ER-positive than in ER-negative tumors and AR-low/FOXA1-low tumors showed high grade, frequent basal-like subtype and worse disease-free survival in ER-positive cancers of public gene dataset, similarly to patient cohort results. The Kaplan-Meier Plotter analysis independently validated patients with both low AR/FOXA1 tumor were significantly associated with worse relapse-free survival in ER-positive cancers. This study suggests that distinctive clinicopathological features according to AR and FOXA1 are determined and a lack of both biomarkers is an independent poor prognostic factor in ER-positive tumors.

## INTRODUCTION

Recent attention has focused on the emerging roles of androgen receptor (AR) not only as a prognostic and predictive factor, but also as a therapeutic target in breast cancer patients [1, 2]. A systematic review and meta-analysis showed that positive AR expression was significantly associated with better survival of patients with early breast cancer irrespective of estrogen receptor (ER) status [3]. However, *in vitro* evidence partly supported clinical studies and AR showed antiproliferative activity in only ER-positive breast cancers but rather AR signaling promoted tumor growth in ER-negative and human epidermal growth factor receptor 2 (HER2)-positive breast tumors [1, 4]. Furthermore, Lehmann et al. [5] identified six subtypes of triple-negative breast cancer (TNBC), one of them being a luminal androgen receptor subtype with distinct features among diversely heterogeneous TNBCs [6]. The clinical or biological impact of AR has not been clearly defined, therefore, additional approaches are necessary to clarify the various roles of AR and its control mechanisms according to ER status.

Forkhead box A1 (FOXA1), initially discovered as hepatocyte nuclear factor 3α (HNF3α), is a member of the FOX family transcription factors [7]. Because of a lack of the basic amino acids in FOXAs for chromatin compaction, binding of FOXAs to nucleosomes creates an open chromatin configuration that can recruit other transcriptional regulators [8, 9]. Thus, FOXA1 belongs to a ‘pioneering factor’ [10]. Recent meta-analyses of breast cancers demonstrated that high FOXA1 levels were positively correlated with ER-positive and progesterone receptor (PR)-positive tumors [11]. Patients with high FOXA1 expression showed better disease-free survival (DFS) and overall survival (OS) [12]. A study by Hurtado et al. [13] supported that FOXA1 played a key role in differentially influencing interactions between ER and chromatin. Genetic analysis of invasive lobular carcinomas, which were predominantly categorized as the luminal A subtype, exhibited recurrent FOXA1 mutations and correlation with high FOXA1 activity [14]. The data concluded that FOXA1 was closely associated with the ER signaling pathway and suggested that FOXA1 may explain heterogeneous features of hormone receptor-positive tumors.

In contrast, molecular apocrine breast tumors are characterized by apocrine histopathological features, ER-negativity, AR-positivity, and HER2 amplification [15, 16]. They have AR-driven, hormonally regulated transcriptional activities mediated by FOXA1, similar to ER-mediated transcription in luminal subtype breast cancers [17]. An ancillary immunohistochemistry (IHC) study of AR and FOXA1 in 592 TNBCs from the UNICANCER PACS08 adjuvant multicenter trial suggested that co-expression of both markers seems to be associated with distinct clinicopathological features of luminal tumors compared to other TNBCs [18]. These findings implied a close molecular connection between AR and FOXA1, however, the clear genetic or clinical implications of these biomarkers on tumor biology and patient prognosis have not been fully explained according to ER status of breast cancer, especially in ER-positive tumors.

The purpose of the present study was to explore the genetic expression patterns and associations between AR and FOXA1 by ER status determined from web-based breast cancer datasets. Next, it was to examine the influence among biomarkers through *in vitro* ER-positive cell lines studies. Finally, the present study aimed to investigate and validate clinicopathological characteristics and survival outcomes according to combined AR and FOXA1 protein and mRNA status in mainly ER-positive patients using clinical data of a single institution and public datasets.

## RESULTS

### Web-based bioinformatics analysis

First, the mRNA expression status of ESR1, AR, and FOXA1 was explored on the cBioPortal website. Genetic alteration of AR and FOXA1 was noted in 4% and 11% of total cases, respectively (Supplementary Figure 1A). Queried gene set was changed in 245 (12.4%) samples. There was no alteration in ESR1 mRNA and only mRNA downregulation was presented in FOXA1. The Molecular Taxonomy of Breast Cancer International Consortium (METABRIC) dataset was able to add a clinical attribute track and when the PAM50 subtype was applied, AR and FOXA1 were mainly altered in the basal subtype. When the mutual exclusivity or co-occurrence of alterations among these 3 biomarkers was investigated, co-occurrence of alterations could be only calculated between AR and FOXA1 because there was no alteration in ESR1 mRNA. A significant tendency towards co-occurrence between AR and FOXA1 was determined in all queried samples (*p* < 0.001; log odds ratio = 2.474). Supplementary Figures 1B-1D show the positive associations of mRNA expression among ESR1, AR, and FOXA1. Among the 3 genes, the positive coefficient was the highest between ESR1 and FOXA1, subsequently between AR and FOXA1, and followed by ESR1 and AR.

### *In vitro* cell lines study

**Figure 1:**
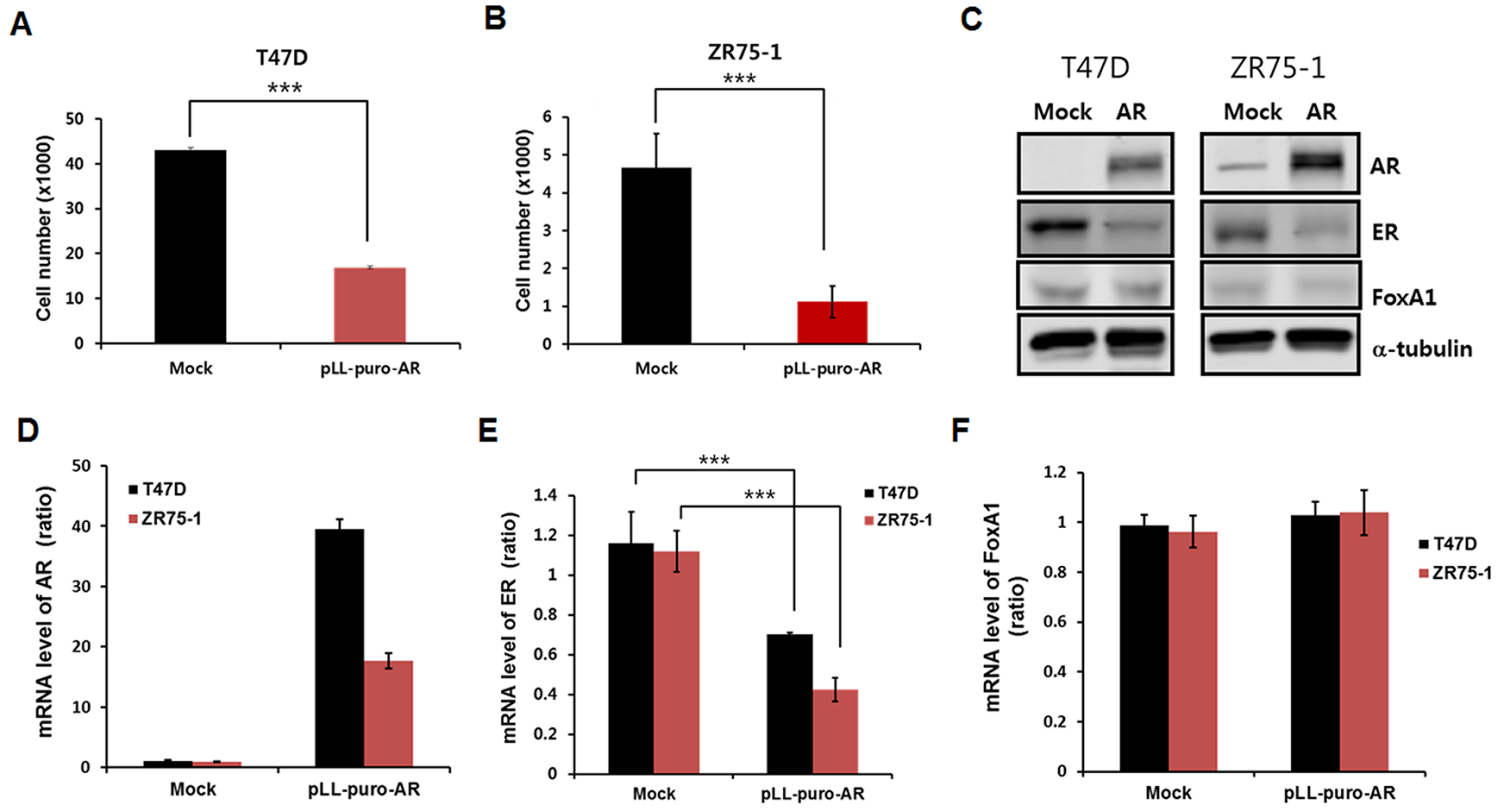
Cell proliferation and mRNA levels using T47D and ZR75-1 cell lines Cell proliferation of T47D and ZR75-1 cell lines decreases by lentiviral overexpression of AR. Empty vector, pLL-CMV-puro was utilized for lentivirus production as a mock control. Stable AR overexpression of **(A)** T47D and **(B)** ZR75-1 cells decreased the number of cells at day 6. **(C)** Protein levels of AR, ER, FOXA1 and α-tubulin are shown by Western blot analysis. α-tubulin was detected as a loading control. Levels of AR, ER, and FOXA1 mRNA in T47D and ZR75-1 cell lines are presented. **(D)** mRNA levels of AR were measured by real-time RT-PCR analysis as described in Materials and Methods. **(E)** Overexpression of AR significantly decreased mRNA levels of ER. **(F)** No effects of AR overexpression on mRNA levels of FOXA1 were observed. Data are presented as the mean ± SD. Two-sample *t*-test, ***p < 0.001.

To investigate the association between AR and FOXA1 in ER-positive tumors, an *in vitro* study was performed using T47D and ZR75-1 breast cancer cell lines. As shown in Figure 1A and 1B, stable cell lines overexpressing AR in both T47D and ZR75-1 exhibited significant decrease in cell proliferation compared with negative control (mock) cells. Notably, Western blot and real-time RT-PCR analyses showed reduced expression levels of ER protein and mRNA in AR-overexpressing cell lines, which suggested that downregulation of ER expression might affect cell proliferation (Figures 1C-1E). Next, in order to examine whether FOXA1 could be altered by AR overexpression or not, protein and mRNA level of FOXA1 were checked. However, FOXA1 expression was not significantly changed by overexpression of AR (Figures 1C and 1F).

**Figure 2:**
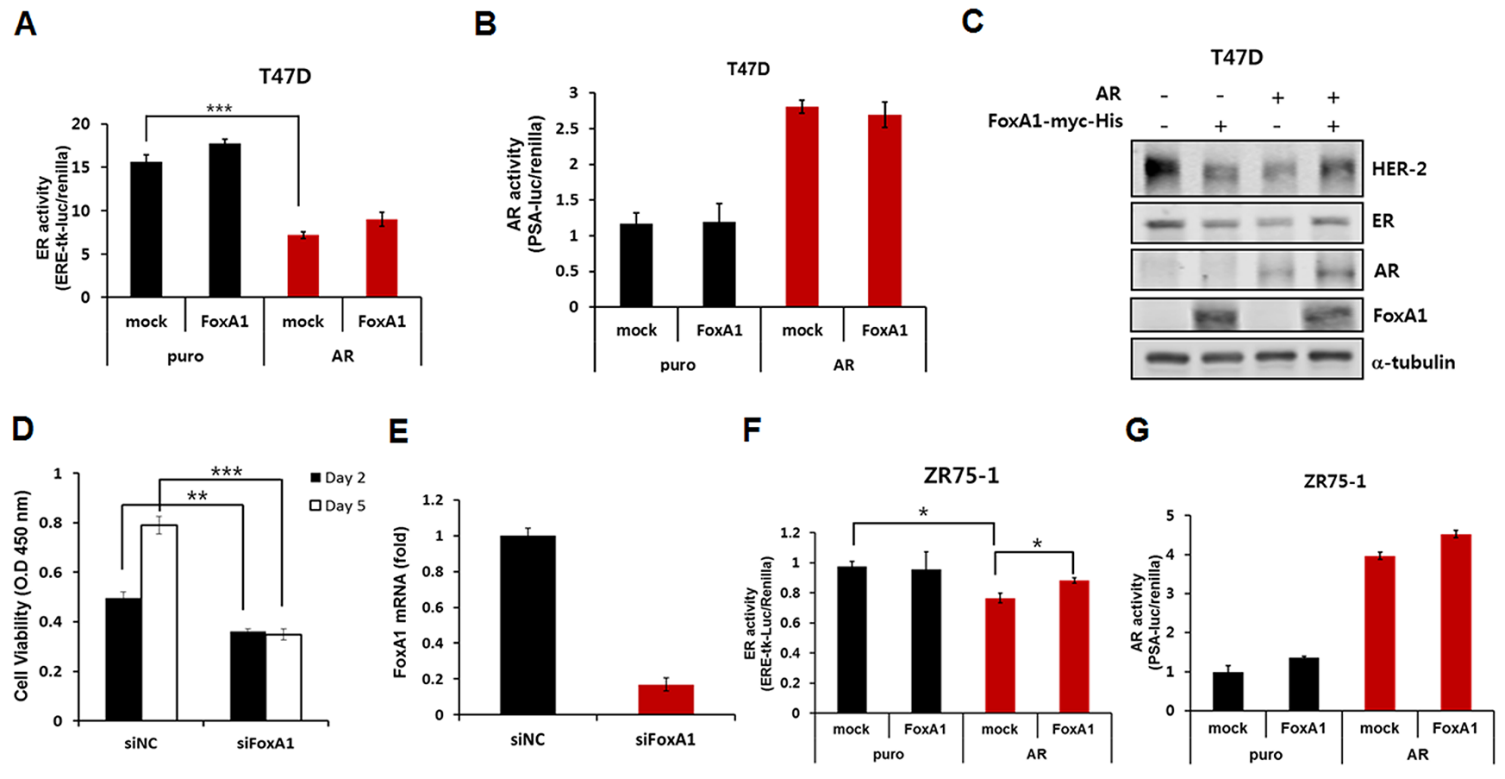
Effects of FOXA1 overexpression on ER activity **(A)** ERE-tk-luciferase activity and **(B)** PSA-luciferase activity was normalized by the *Renilla* expression level in T47D cell line. **(C)** Western blot analysis of HER2, ER, AR and FOXA1 expression in FOXA1- and/or AR-overexpressing T47D cell line. Expression of ɑ-tubulin was analyzed as a loading control. **(D)** Viability of T47D cells was measured at day 2 and day 5 after the treatments of siRNA against non-targeted sequence and FOXA1. **(E)** FOXA1 mRNA levels were evaluated after treatments of siRNA by quantitative real-time RT-PCR analysis. **(F)** ERE-tk-luciferase activity and **(G)** PSA-luciferase activity was normalized by the *Renilla* expression level in ZR75-1 cell line. Data are presented as the mean ± SD. Two-sample t-test, *p < 0.05, **p < 0.005, ***p < 0.001.

Next, the effects of FOXA1 overexpression on ER activity were compared in mock- and AR-overexpressing T47D cell lines. As shown in Figures 2A-2C, overexpression of FOXA1 in these cell lines had no effect on ER and AR activity. However, knockdown of FOXA1 resulted in a significant reduction of cellular viability on day 5 (Figures 2D-2E), suggesting that FOXA1 has essential roles for viability of the ER-positive tumor cell lines, although there were no direct effects on ER and AR activities. Experiments with ZR75-1 showed similar results (Figures 2F and 2G). ER activity was higher in both AR and FOXA1 overexpressing cell lines than in AR alone overexpressed cells, however, lower than in mock or FOXA1 alone overexpressed cell lines (Figures 2A and 2F).

### Clinicopathological characteristics and patient survival in the tissue microarray (TMA) study

**Figure 3:**
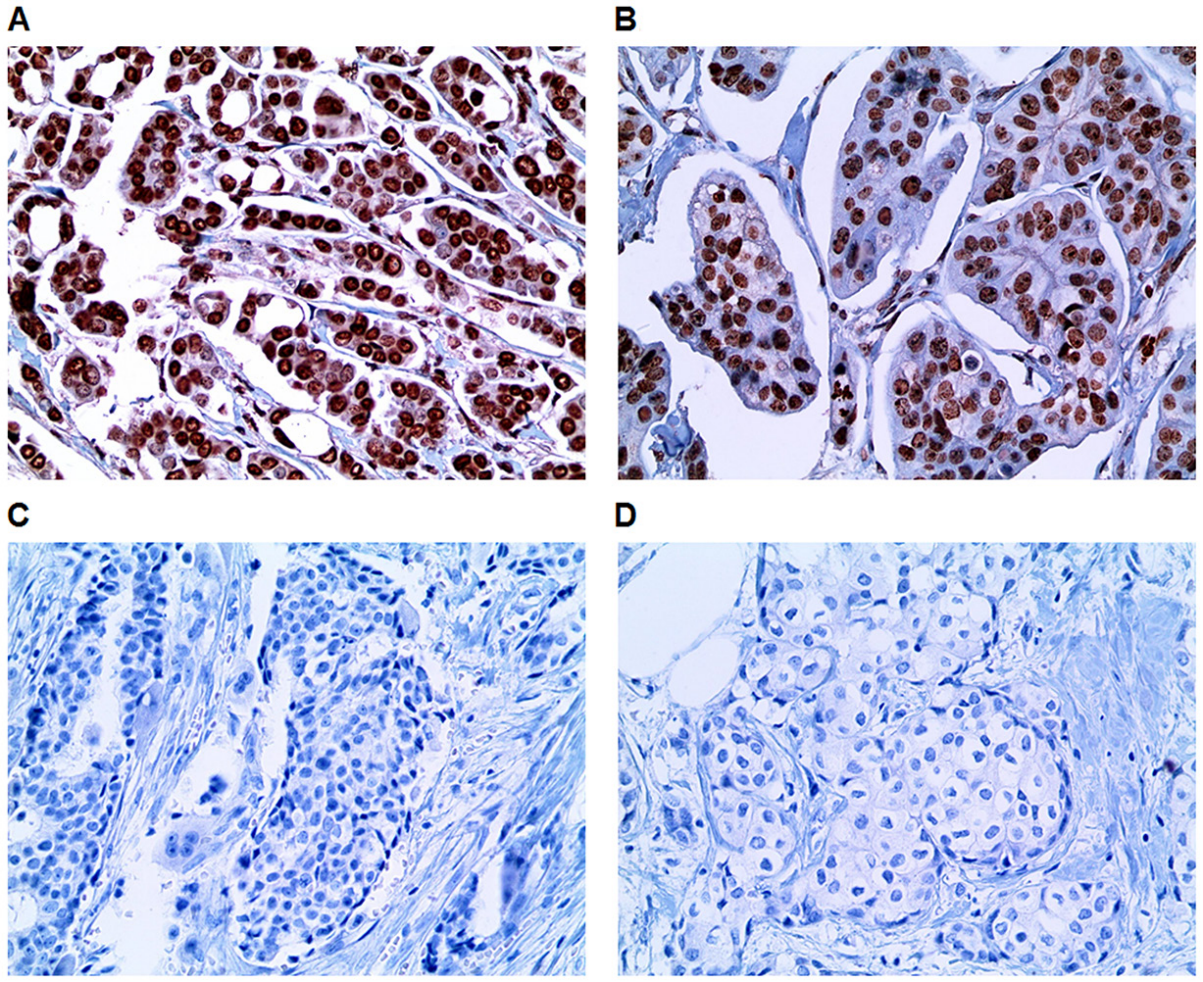
Representative immunohistochemical staining of the TMA study Photographs show positive immunohistochemical expression of **(A)** AR and **(B)** FOXA1 and negative expression of **(C)** AR and **(D)** FOXA1 in the TMA slides (x400, H&E stain).

Using breast cancer patient population treated at a single institute, the prognostic value of immunohistochemically determined AR and FOXA1 status was investigated. In all patients, AR and FOXA1 positivity was 55.8% and 72.1%, respectively (Figure 3). AR positivity was significantly associated with FOXA1 positivity (*p* < 0.001) and 384 (44.3%) patients had tumors that were AR and FOXA1 positive. AR and FOXA1 negativity was noted in 143 (16.5%) patients. Supplementary Table 1 shows the clinicopathological characteristics according to AR and FOXA1 status in all patients. AR(+)/FOXA1(+) tumors were significantly associated with small tumor size, lower TNM stage, grade I/II, hormone receptors-positive expression, and luminal A subtype. Patients with AR(+)/FOXA1(-) tumors showed the highest frequency of HER2-positive, low Ki-67 proliferative index tumors. Treatment patterns were not significantly different among groups except for endocrine therapy. When examining the clinicopathological characteristics of ER-positive tumors based on AR and FOXA1 status, similar trends were observed between the AR(+)/FOXA1(+) group and histopathological parameters, including smaller size, node-negative disease, lower stage, higher grade I/II, and PR-positive expression (Table 1). The AR(-)/FOXA1(-) group frequently showed node metastasis, high grade, PR-negative expression, and high Ki-67 proliferation.

**Table 1:** Clinicopathological characteristics according to AR and FOXA1 expression in ER-positive breast cancer patients from the TMA study

Factor	AR(+)/ FOXA1(+) (n = 356, %)	AR(+)/ FOXA1(-) (n = 68, %)	AR(-)/ FOXA1(+) (n = 141, %)	AR(-)/ FOXA1(-) (n = 60, %)	*p*-value
Age (years)					
≤ 50	230 (64.6)	40 (58.8)	101 (71.6)	38 (63.3)	0.269
> 50	126 (35.4)	28 (41.2)	40 (28.4)	22 (36.7)	
Tumor stage					
pT1	199 (55.9)	32 (47.1)	51 (36.2)	26 (43.3)	< 0.001
pT2-4	157 (44.1)	36 (52.9)	90 (63.8)	34 (56.7)	
Node stage					
pN0	189 (53.1)	32 (47.1)	59 (41.8)	21 (35.0)	0.019
pN1-3	167 (46.9)	36 (52.9)	82 (58.2)	39 (65.0)	
Histologic grade					
I/II	309 (86.8)	58 (85.3)	109 (77.3)	46 (76.7)	0.030
III	47 (13.2)	10 (14.7)	32 (22.7)	14 (23.3)	
PR					
Positive	306 (86.0)	59 (86.8)	112 (79.4)	41 (68.3)	0.004
Negative	50 (14.0)	9 (13.2)	29 (20.6)	19 (31.7)	
HER2					
Negative	284 (79.8)	54 (79.4)	104 (73.8)	52 (86.7)	0.203
Positive	72 (20.2)	14 (20.6)	37 (26.2)	8 (13.3)	
Ki-67 (n = 623)					
< 15%	326 (91.8)	65 (97.0)	122 (86.5)	45 (75.0)	< 0.001
≥ 15%	29 (8.2)	2 (3.0)	19 (13.5)	15 (25.0)	
Type of surgery					
BCS	106 (29.8)	24 (35.3)	33 (23.4)	10 (16.7)	0.053
TM	250 (70.2)	44 (64.7)	108 (76.6)	50 (83.3)	
Radiation therapy					
Not done	187 (52.5)	32 (47.1)	77 (54.6)	35 (58.3)	0.609
Done	169 (47.5)	36 (52.9)	64 (45.4)	25 (41.7)	
Chemotherapy					
Not done	55 (15.4)	11 (16.2)	12 (8.5)	10 (16.7)	0.191
Done	301 (84.6)	57 (83.8)	129 (91.5)	50 (83.3)	
Endocrine therapy					
Not done	30 (8.4)	9 (13.2)	20 (14.2)	12 (20.0)	0.031
Done	326 (91.6)	59 (86.8)	121 (85.8)	48 (80.0)	

AR: androgen receptor, FOXA1: forkhead box A1, ER: estrogen receptor, TMA: tissue microarray, PR: progesterone receptor, HER2: human epidermal growth factor receptor 2, BCS: breast conservation surgery, TM: total mastectomy.

**Figure 4:**
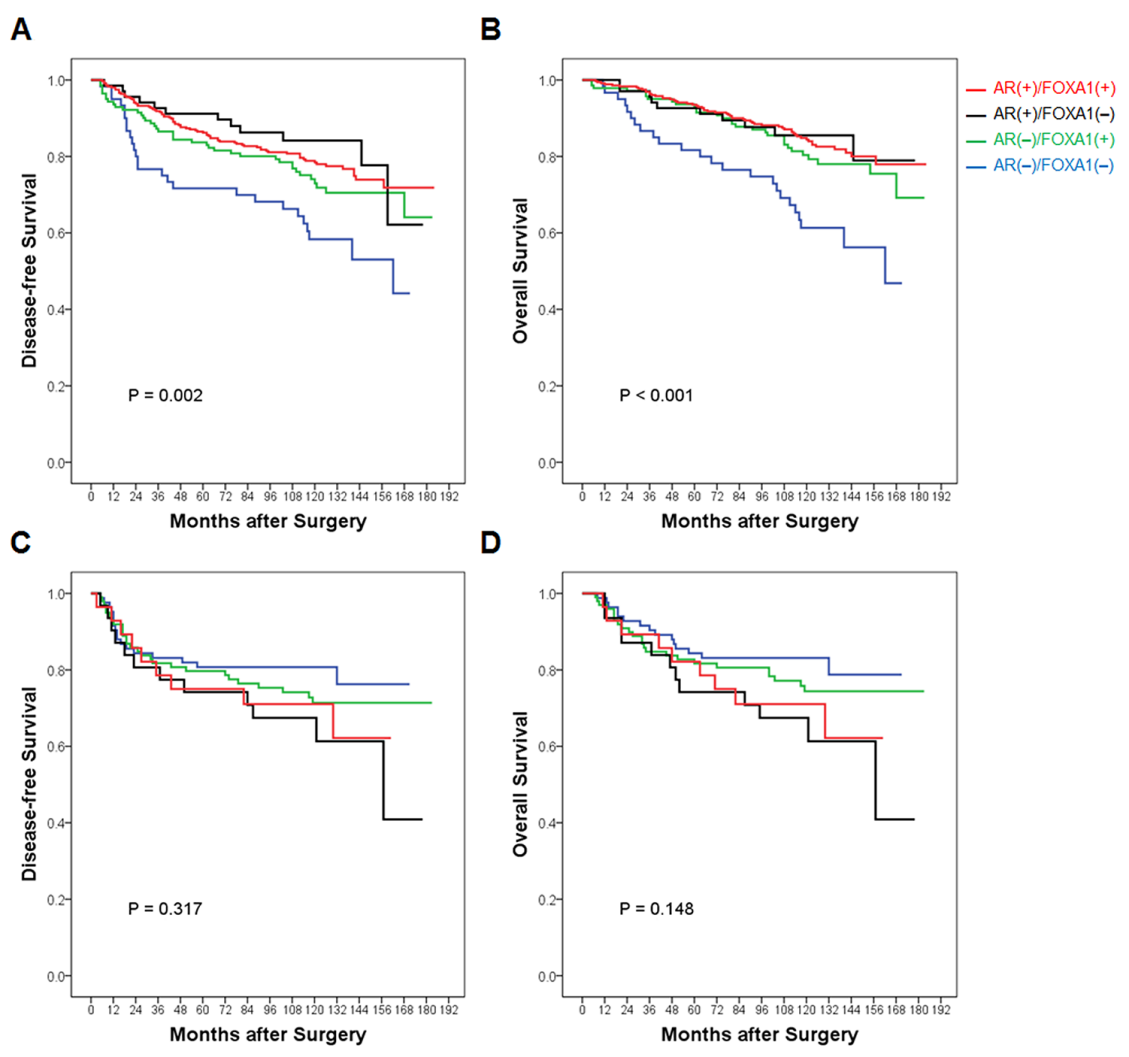
Survival curves according to AR and FOXA1 status stratified by ER expression in the TMA study In patients with **(A** and **B)** ER-positive and **(C** and **D)** ER-negative tumors of the TMA study, respectively, (A and C) disease-free survival and (C and D) overall survival are compared according to AR and FOXA1 expression status. Red line represents AR(+)/FOXA1(+), black line, AR(+)/FOXA1(-), green line, AR(-)/FOXA1(+), and blue line, AR(-)/FOXA1(-) subgroups, respectively. Overall *p*-value is calculated among subgroups and presented in each Kaplan-Meier survival curve.

During mean follow-up periods of 112.8 months [standard deviation (SD) = 39.9], 222 (25.6%) patients had pre-defined events and 183 (21.1%) patients died. DFS and OS curves according to AR and FOXA1 status showed no statistically significant prognostic value in all patients (Supplementary Figure 2). However, when survival stratified by ER status was analyzed, AR(-)/FOXA1(-) tumors showed significantly worse DFS and OS than either AR(+) or FOXA1(+) tumors in ER-positive patients. Among AR(+) and/or FOXA1(+) tumors, there was no statistical difference in survival according to AR/FOXA1 status in patients with ER-positive tumors (Figures 4A and 4B). No statistical difference in survival among groups was demonstrated in ER-negative tumors (Figures 4C and 4D). Rather, AR(-)/FOXA1(-) tumors rather showed a trend toward better survival in the TMA study.

In patients with ER-positive tumors, multivariate analysis revealed that AR(-)/FOXA1(-) tumors were independently poor prognostic factors for DFS and OS when age at diagnosis, tumor and node stage, histologic grade, HER2, Ki-67, and use of chemotherapy and endocrine therapy were adjusted (Table 2). Node metastasis, HER2-positivity, and absence of chemotherapy were also significantly associated with increased risk of poor DFS and OS in the TMA study.

**Table 2:** Multivariate analysis for survival of ER-positive breast cancer patients in the TMA study

Factors	Disease-free survival			Overall survival		
	HR	95% CI	*p*-value	HR	95% CI	*p*-value
AR/FOXA1						
AR(-)/FOXA1(-)	Ref				Ref	
AR(-)/FOXA1(+)	0.579	0.348 - 0.964	0.036	0.451	0.261 - 0.780	0.004
AR(+)/FOXA1(-)	0.392	0.195 - 0.790	0.009	0.352	0.166 - 0.749	0.007
AR(+)/FOXA1(+)	0.552	0.349 - 0.875	0.011	0.417	0.255 - 0.682	< 0.001
Age (≤ 50 years)	0.921	0.651 - 1.303	0.641	0.754	0.510 - 1.114	0.157
Tumor stage (pT2-4)	1.307	0.922 - 1.852	0.133	1.451	0.977 - 2.155	0.065
Node stage (pN1-3)	2.846	1.912 - 4.237	< 0.001	2.642	1.696 - 4.113	< 0.001
Histologic grade (III)	1.000	0.653 - 1.530	0.999	0.840	0.512 - 1.379	0.491
HER2 (positive)	1.609	1.111 - 2.331	0.012	1.655	1.101 - 2.490	0.016
Ki-67 (≥15%)	1.295	0.767 - 2.184	0.333	1.196	0.657 - 2.177	0.558
Chemotherapy (not done)	2.390	1.441 - 3.963	0.001	2.282	1.316 - 3.955	0.003
Endocrine therapy (not done)	1.132	0.703 - 1.822	0.611	1.211	0.713 - 2.057	0.478

HR: hazard ratio, CI: confidence interval, Ref: reference.

### Clinicopathological characteristics of the METABRIC datasets

**Figure 5:**
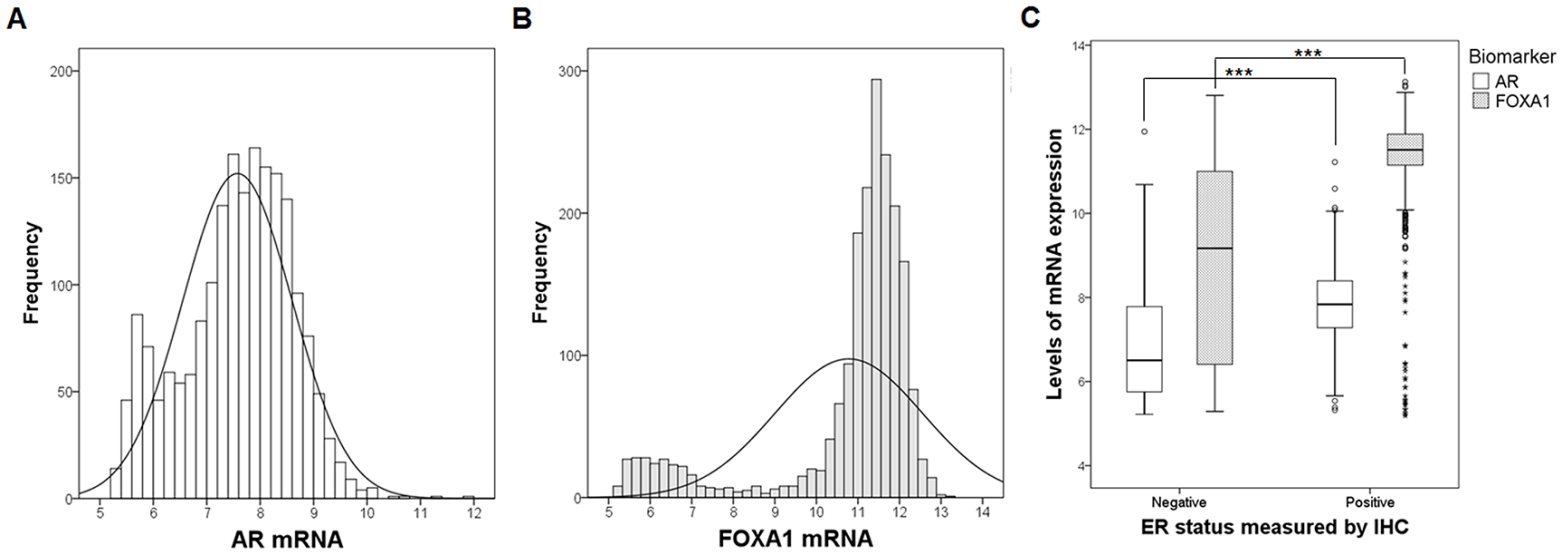
Frequencies and levels of AR and FOXA1 mRNA expression Using the METABRIC dataset, frequencies of **(A)** AR and **(B)** FOXA1 mRNA expression are shown. **(C)** Levels of mRNA expression according to ER status are presented using box plot with whiskers. White box represents AR and dark box shows FOXA1. Mean levels of each AR and FOXA1 are compared using two-sample t-test between ER-positive and ER-negative samples. ***p < 0.001.

Using data of mRNA levels and clinical attributes from the METABRIC dataset, AR and FOXA1 mRNA expression patterns and clinicopathological characteristics according to AR/FOXA1 status were investigated. The median values of AR and FOXA1 mRNA were 7.57 [interquartile range (IQR), 1.39] and 11.37 (IQR, 0.96), respectively. The frequencies of AR and FOXA1 mRNA levels are shown in Figure 5A and 5B, respectively. The distribution of mRNA expression was unimodal for AR, but bimodal for FOXA1. The clinical parameters of ER status by IHC were available in 1,923 patients of the METABRIC dataset. The mean AR mRNA levels were 7.80 (SD = 0.84) in ER-positive samples and 6.82 (SD = 1.22) in ER-negative samples. The mean FOXA1 mRNA values were 11.40 (SD = 0.92) in ER-positive cancers and 8.72 (SD = 2.32) in ER-negative cancers. AR and FOXA1 mRNA levels were significantly higher in ER-positive tumors than in ER-negative tumors (Figure 5C). The correlation between AR and FOXA1 mRNA expression in the METABRIC dataset was moderately positive (Pearson *r* = 0.424; *p* < 0.001) in ER-positive cancers and strongly positive (Pearson *r* = 0.777; *p* < 0.001) in ER-negative cancers. Since the SD range in consideration with mean value was relatively wider in ER-negative than in ER-positive tumors, and most samples with high AR mRNA levels exhibited high FOXA1 expression in ER-positive cancers; therefore, the correlation coefficient was higher in ER-negative tumors.

The lower quartile cutoff values for defining high versus low mRNA expression of biomarkers were determined 6.93 for AR and 10.81 for FOXA1 from the METABRIC dataset. By these cutoff points, the number of AR-high/FOXA1-high, AR-high/FOXA1-low, AR-low/FOXA1-high, and AR-low/FOXA1-low cases was 1,303 (66.5%), 170 (8.7%), 168 (8.6%), and 317 (16.2%), respectively. In the whole population of the METABRIC dataset, AR-low/FOXA1-low tumors were significantly associated with age at diagnosis ≤ 50 years, high grade, ER-negativity, PR-negativity, HER2-negativity, and the basal-like subtype (Supplementary Table 2). AR-high/FOXA1-low subgroup showed the highest frequency of stage III disease, HER2-positive tumors, and the HER2-enriched subtype (Supplementary Table 2). The clinicopathological characteristics determined by to AR and FOXA1 status in ER-positive breast cancer patients of the METABRIC dataset are presented in Table 3. Similarly, in ER-positive tumors, AR-low/FOXA1-low cases were significantly associated with high grade, PR-negativity, the basal-like subtype, and chemotherapy administration. AR-low/FOXA1-low tumors also showed higher advanced stage and HER2-negativity, but without statistical significance.

**Table 3:** Clinicopathological characteristics according to AR and FOXA1 mRNA status in ER-positive breast cancer patients from the METABRIC dataset

Factor	AR-high/FOXA1-high(n, %)	AR-high/FOXA1-low(n, %)	AR-low/FOXA1-high(n, %)	AR-low/FOXA1-low(n, %)	*p*-value
Age (yrs)					
≤ 50	172 (14.7)	11 (11.3)	33 (22.3)	14 (19.4)	0.048
> 50	996 (85.3)	86 (88.7)	115 (77.7)	58 (80.6)	
TNM stage					
Stage I	336 (31.5)	28 (29.8)	60 (40.8)	26 (36.1)	0.085
Stage II	685 (59.0)	54 (57.4)	78 (53.1)	35 (48.6)	
Stage III	110 (9.5)	12 (12.8)	9 (6.1)	11 (15.3)	
Histologic grade					
I/II	701 (62.8)	44 (47.3)	79 (54.5)	34 (47.2)	0.001
III	415 (37.2)	49 (52.7)	66 (45.5)	38 (52.8)	
PR^*^					
Positive	823 (70.5)	48 (49.5)	81 (54.7)	15 (20.8)	< 0.001
Negative	345 (29.5)	49 (50.5)	67 (45.3)	57 (79.2)	
HER2^*^					
Negative	1,081 (92.6)	86 (88.7)	135 (91.2)	68 (94.4)	0.457
Positive	87 (7.4)	11 (11.3)	13 (8.8)	4 (5.6)	
PAM50 subtype					
Luminal A	613 (52.7)	19 (20.0)	56 (37.8)	4 (5.6)	< 0.001
Luminal B	373 (32.0)	20 (21.1)	62 (41.9)	6 (8.3)	
HER2	74 (6.4)	15 (15.8)	15 (10.1)	9 (12.5)	
Basal	9 (0.8)	14 (14.7)	5 (3.4)	30 (41.7)	
Normal	95 (8.2)	27 (28.4)	10 (6.8)	23 (31.9)	
Type of surgery					
BCS	459 (39.5)	38 (40.9)	70 (47.6)	30 (42.9)	0.296
TM	702 (60.5)	55 (59.1)	77 (52.4)	40 (57.1)	
Radiotherapy					
Not done	510 (43.7)	32 (33.0)	57 (38.5)	26 (36.1)	0.097
Done	658 (56.3)	65 (67.0)	91 (61.5)	46 (63.9)	
Chemotherapy					
Not done	1,073 (91.9)	92 (94.8)	128 (86.5)	55 (76.4)	< 0.001
Done	95 (8.1)	5 (5.2)	20 (13.5)	17 (23.6)	
Hormone therapy					
Not done	311 (26.6)	24 (24.7)	37 (25.0)	17 (23.6)	0.902
Done	857 (73.4)	73 (75.3)	111 (75.0)	55 (76.4)	

*Positive criteria of PR and HER2 were defined as expression status in the METABRIC dataset.

### Survival analysis of the METABRIC dataset

**Figure 6:**
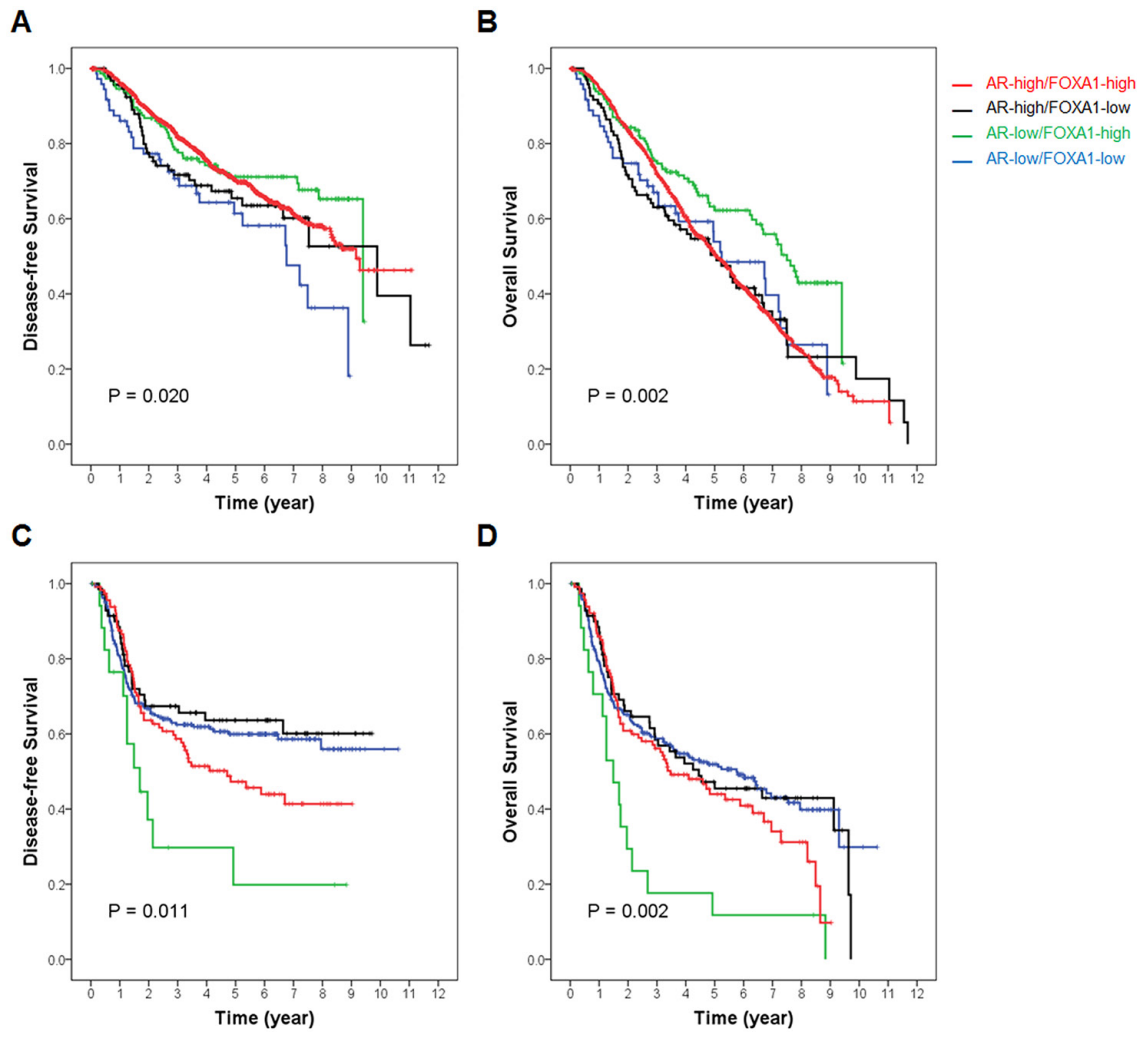
Survival curves according to AR and FOXA1 status stratified by ER expression in the METABRIC dataset In patients with **(A** and **B)** ER-positive and **(C** and **D)** ER-negative tumors of the METABRIC dataset, respectively, (A and C) disease-free survival and (C and D) overall survival are compared according to AR and FOXA1 expression level. Red line represents AR-high/FOXA1-high, black line, AR-high/FOXA1-low, green line, AR-low/FOXA1-high, and blue line, AR-low/FOXA1-low subgroups, respectively. Overall *p*-value is calculated among subgroups and presented in each Kaplan-Meier survival curve.

The METABRIC dataset indicated the mean follow-up duration was 125.6 months (n = 1,958; SD = 76.1) and recurrent or progressive events and deaths were in 32.6% and 57.8% of patients, respectively. Supplementary Figure 3 shows DFS and OS curves according to AR and FOXA1 mRNA status. Compared to other groups, the AR-low/FOXA1-low group showed the worst 5-year DFS with statistical significance in the whole population. The OS curve demonstrated no statistical significance. Similarly, in ER-positive patients, the AR-low/FOXA1-low group showed the worst 5-year DFS (*p* = 0.020) and the AR-low/FOXA1-high group exhibited the best statistically significant 5-year OS (*p* = 0.002). However, the AR-low/FOXA1-high group presented the worst 5-year DFS (*p* = 0.011) and 5-year OS (*p* = 0.002) in ER-negative tumors (Figure 6).

To investigate the prognostic roles of AR and FOXA1 status in ER-positive breast cancers, multivariate analysis was performed using clinical variables of the METABRIC dataset (Table 4). The AR-low/FOXA1-low group was determined to be a significantly poor prognostic factor than the AR-low/FOXA1-high and AR-high/FOXA1-high groups for DFS and the AR-low/FOXA1-high group for OS when age, stage, grade, HER2, and use of chemotherapy and hormone therapy were adjusted.

**Table 4:** Multivariate analysis for survival of ER-positive breast cancer patients in the METABRIC dataset

Factors	Disease-free survival			Overall survival		
	HR	95% CI	*p*-value	HR	95% CI	*p*-value
AR/FOXA1						
AR-low/FOXA1-low	Ref			Ref		
AR-low/FOXA1-high	0.566	0.351 - 0.912	0.019	0.661	0.440 - 0.995	0.047
AR-high/FOXA1-low	0.775	0.468 - 1.283	0.322	0.992	0.651 - 1.511	0.969
AR-high/FOXA1-high	0.648	0.443 - 0.950	0.026	0.996	0.713 - 1.392	0.982
Age (≤ 50 years)	0.664	0.491 - 0.896	0.008	0.407	0.314 - 0.527	< 0.001
TNM stage						
Stage I	Ref			Ref		
Stage II	1.654	1.287 - 2.126	< 0.001	1.604	1.348 - 1.909	< 0.001
Stage III	3.940	2.830 - 5.484	< 0.001	2.766	2.148 - 3.562	< 0.001
Histologic grade (III)	1.561	1.282 - 1.901	< 0.001	1.239	1.075 - 1.429	0.003
HER2 (positive)	1.871	1.395 - 2.510	< 0.001	1.474	1.156 - 1.880	0.002
Chemotherapy (not done)	0.672	0.489 - 0.924	0.015	0.840	0.636 - 1.110	0.220
Endocrine therapy (not done)	1.067	0.826 - 1.379	0.619	0.960	0.802 - 1.148	0.651

### Survival analysis of the kaplan-meier (KM) plotter

**Figure 7:**
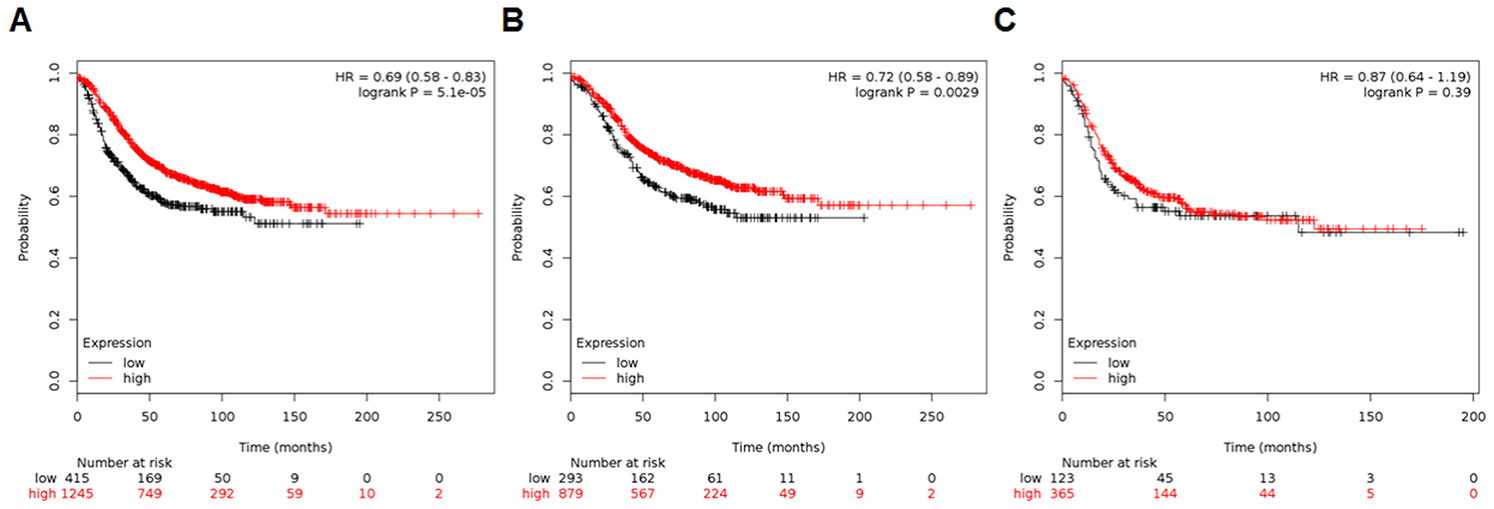
Relapse-free survival curves using a multigene classifier of the KM Plotter Plots are generated according to AR and FOXA1 levels in **(A)** all patients, **(B)** ER-positive cancers, and **(C)** ER-negative tumors from the KM Plotter website (http://kmplot.com/analysis).

Finally, the KM Plotter analysis was performed to validate the prognostic value of combined AR and FOXA1 mRNA status. A multigene classifier uses the mean expression of the selected genes, and a new value [(gene X1 + gene X2 + ··· + gene Xn)/n] is computed for survival analysis of the KM Plotter. Figure 7A shows relapse-free survival curves according to AR and FOXA1 levels in 1,660 patients with available data. Patients with low AR/FOXA1 expression levels demonstrated significantly lower survival in all patients (HR, 0.69; *p* < 0.001). This statistical significance was maintained in only 1,172 ER-positive tumors (HR, 0.72; *p* = 0.003; Figure 7B) but not in 488 ER-negative cancers (HR, 0.87; *p* = 0.392; Figure 7C).

The KM Plotter provides subgroup analyses according to the intrinsic subtype based on the 2013 St. Gallen criteria using the expression of ESR1, HER2, and MKI67 as follows; luminal A (ESR1+/HER2–/MKI67 low), luminal B (ESR1+/HER2–/MKI67 high and ESR1+/HER2+), HER2-enriched (ESR1–/ HER2+), and basal subtype (ESR1–/HER2–) [19, 20]. Stratification by the intrinsic subtypes is presented in Supplementary Figure 4. Patients with low AR/FOXA1 expression exhibited poor relapse-free survival in 783 luminal A subtype tumors (HR, 0.65; 95% CI, 0.49–0.86; log-rank *p* = 0.002), but not in other subtypes (HR, 1.12; 95% CI, 0.77–1.63; log-rank *p* = 0.55 for 389 luminal B, HR, 1.3; 95% CI, 0.72–2.33; log-rank *p* = 0.39 for 149 HER2-enriched, and HR, 1.04; 95% CI, 0.71–1.53; log-rank *p* = 0.84 for 339 basal subtypes).

## DISCUSSION

An exploration of the web-based genetic analysis in the present study showed that approximately 10% of breast cancers were altered in ESR1, AR, or FOXA1 genes and generally changes in genes concurrently occurred even though the frequency was low. The roles of changes in the AR or FOXA1 genes have not been much studied in female breast cancers. However, this study showed a close correlation between AR and FOXA1 expression levels. Clinically undetermined genetic networks between these markers have been proposed in ER-negative cancers, suggesting combined biomarker studies may be critical [17, 21]. Our *in vitro* model presenting no significant change in FOXA1 by AR overexpression and vice versa and multivariate analysis demonstrating statistical significance of AR and FOXA1 status suggested that combined biomarker was independently significant in ER-positive cancers, although the number of cases with alteration in AR and FOXA1 was very small.

Peters et al. [4] provided supporting data that growth inhibition of ER-positive breast cancer by androgens was directly mediated by AR and was derived from inhibition of the ER signaling pathway rather than via activation of AR-regulated target genes. Our *in vitro* study also confirmed that overexpression of AR induced downregulation of ER expression and cellular proliferation. A subsequent cistrome study demonstrated that AR signaling was less likely to rely on FOXA1 than ER colocalization in ZR75-1 cells [22]. Approximately 20% of all peaks and > 60% of high-stringency sites showed a direct overlap between ER and FOXA1 binding sites. However, only 8% of all peaks and about 35% of high-stringency sites overlapped between AR and FOXA1 binding sites [13, 22]. These findings suggest that each AR and FOXA1 exerts its function in specific cellular situations. Knockdown of FOXA1 induced marked loss of ER-positive cell viability on day 5 as shown in Figure 2. These findings suggested that FOXA1, as a pioneering transcription factor in downstream of the ER signaling pathway, contributes tumor survival, as shown as a lineage-specific oncogene in luminal cancer cell lines [13, 23]. Recently, the dual roles of FOXA1 in breast cancer as a growth stimulator and inhibitor have been considered controversial [9, 12, 24]. A comprehensive analysis and an individualized interpretation may be required for understanding the role of FOXA1.

Approximately 10–20% of all cases showed negative protein expression or low mRNA levels of both AR and FOXA1 in our study. These cases were significantly associated with aggressive tumor features such as ER-negative, PR-negative, TNBC, basal subtype, high grade, and high Ki-67 labelling index. These features were maintained in patients with ER-positive cancer. Habashy et al. [25] demonstrated that while a combined analysis was not performed, negative FOXA1 was significantly associated with negative ER, AR, and PR expression in both whole series and ER-positive cohorts. On the contrary, 15.2% of 460 patients with TNBC showed AR(+)/FOXA1(+) tumors, which were associated with frequent lobular histology, older age at diagnosis, lower nuclear grade, and less presentation of lymphocytic infiltration, pushing margin, syncytial architecture, and central fibrosis or necrosis from the UNICANCER PACS08 trial [18]. Breast cancer cell lines with molecular apocrine features showed a significant functional cross-talk between AR and HER2 that involved FOXA1 activity [26]. These suggested that loss of AR and FOXA1 in ER-positive breast cancers and gain of AR and FOXA1 in ER-negative tumors were possible markers of distinct biological phenotypes.

Interestingly, a half of the apocrine carcinomas overexpressed the HER2 protein [27]. Regarding the association of AR/FOXA1 status with HER2 in the present study, TMA analysis and public dataset showed no statistical significance in ER-positive cancers, although decreased HER2 protein was noted by experiments overexpressing AR or FOXA1 (Figure 2C). In ER-negative patients, however, positive HER2 was 67.9% of AR(+)/FOXA1(+), 67.7% of AR(+)/FOXA1(-), 23.2% of AR(-)/FOXA1(+), and 21.7% of AR(-)/FOXA1(-) tumors in the TMA study (*p* < 0.001). In ER-negative samples of the METABRIC dataset, positive HER2 was 57.9% of AR-high/FOXA1-high, 47.1% of AR-high/FOXA1-low, 44.4% of AR-low/FOXA1-high, and 6.8% of AR-low/FOXA1-low tumors (*p* < 0.001). Although the proportion of HER2- positive cases in ER-negative tumors was different among datasets, AR- or FOXA1-positive patients showed higher HER2-positive tumors. Therefore, further studies are necessary to understand the clinical implications of these networks in ER-negative breast cancers.

As a prognostic marker, AR is consistently reported to be associated with better survival outcomes [3, 28, 29]. Although somewhat conflicting results have been suggested, many studies demonstrate FOXA1 expression as a good prognostic factor [11, 12, 30, 31]. However, since AR and FOXA1 could be closely connected as shown in protein and mRNA expression status analyses of the present study, the clinical impact of AR and FOXA1 on survival should be analyzed considering ER status. However, only a few studies have been conducted. This study demonstrated that patients with negative protein or low mRNA expression of both AR and FOXA1 showed independently poor survival outcomes in ER-positive cancers from the TMA study and the METABRIC dataset. This association was also validated in analyses of the KM Plotter. Therefore, future analysis with longer follow-up periods should be conducted to confirm the hypothesis.

Interestingly, according to analyses of ER-negative tumors using the METABRIC dataset, 18 (4.1%) cases with AR-low/FOXA1-high tumor showed the worst survival and 114 (26.0%) with AR-high/FOXA1-high presented worse DFS with statistical significance. In the TMA study, AR(-)/FOXA1(-) or AR(-)/FOXA1(+) subgroups demonstrated a trend of better survival in ER-negative patients, although no statistical significance was noted. Additional studies with larger sample sizes should be required to understand the different clinical impact of AR and FOXA1 status on survival in ER-negative breast cancers.

A potential limitation of the present study was inevitably the nonrandomized and retrospective nature of the clinical dataset. Difficulty in handling and manipulation of an *in vitro* study could not find out details of subcellular molecular mechanisms between AR and FOXA1 and many other ER-positive, luminal subtype breast cancer cell lines were not investigated. In addition, methodological problems were key issues. The evaluation and interpretation of immunohistochemical AR and FOXA1 expression were not standardized and the use of TMA tumor blocks with small sized cores may not have been able to represent the results of whole sections. Detection methods and cutoff values of public datasets were varied and arbitrary. Among the genomic profiles on the bioinformatics analysis website, the number of mutations, copy-number alterations, or methylations was not incorporated into the present study and only mRNA expression data were used. Results from the independent datasets could not be used to calculate the associations between protein and mRNA expression levels of biomarkers. Nevertheless, the present study had strengths to explore and validate the undisclosed role of combined AR and FOXA1 status in ER-positive breast cancers using the genetic and clinical datasets with *in vitro* cell lines study.

In conclusion, the present results indicate that AR and FOXA1 are closely associated in breast cancers, and distinctive clinicopathological features are presented in ER-positive tumors according to AR and FOXA1 status. More importantly, loss of or decrease in both AR and FOXA1 expression is an independently significant poor prognostic factor in ER-positive tumors. Since different molecular mechanisms between AR and FOXA1 signaling pathways have been suggested in ER-negative breast cancers, the clinical implications of AR and FOXA1 status on patient prognosis should be further investigated to improve the survival of patients with heterogeneous breast cancers. Therefore, possible therapeutic strategies such as anti-androgens should be examined considering the AR, FOXA1, and ER status in breast cancer patients.

## MATERIALS AND METHODS

### The cBioPortal for cancer genomics

Genomic analysis was performed for investigating the associations between ESR1, AR, and FOXA1 through the cBioPortal for Cancer Genomics (http://www.cbioportal.org), which provides web-based visualization and access to large-scale cancer genomic datasets [32, 33]. The METABRIC dataset was selected for analysis. The dataset included RNA sequencing data and clinicopathological information of 1,980 samples obtained from 1,980 patients (June, 2016) [34]. Expression by U133 microarray was selected to generate an OncoPrint in the cBioPortal website for visualizing the genetic alteration and to investigate the mutual exclusivity or co-occurrence of alterations among biomarkers in all analyzed samples.

Raw data of AR and FOXA1 mRNA expression and clinical information in a dataset were downloaded from the cBioPortal website to explore the association of AR and FOXA1 status with clinicopathological characteristics and survival, mainly in immunohistochemically determined ER-positive breast tumors. The lower quartile cutoff values were arbitrarily selected to determine high and low expression levels of AR and FOXA1. Of 1,980 samples within the METABRIC dataset, cases with stage 0 disease (n = 12) or stage IV disease (n = 10) were excluded from analysis. Upon exclusion, 1,958 samples with stage I–III disease were analyzed for survival in this study.

### The KM plotter

The probability of relapse-free survival according to AR and FOXA1 status including subgroup analyses was calculated using the KM Plotter (http://kmplot.com/analysis) [35]. It is an online tool that allows analysis of the effects of 54,675 genes on survival by using 10,188 cancer samples, which includes 4,142 breast cancer patients with a mean follow-up duration of 69 months (June, 2016). Survival and gene expression data were derived from the Gene Expression Omnibus (Affymetrix microarray only), European Genome- phenome Atlas, and TCGA. The Affymetrix probe set IDs selected were 226197_at for AR and 204667_at for FOXA1 in the present study. Multiple genes were entered through a multigene classifier using the mean expression of selected biomarkers. To analyze the prognostic value of combined AR and FOXA1, the patient samples were split into two groups using the lower quartile as a cutoff value. Hazard ratio (HR) with 95% confidence interval (CI) and log-rank *p*-value were calculated, and survival curves were displayed on the webpage.

### *In vitro* cell lines study

Human breast cancer cell lines (T47D and ZR75-1) were obtained from the American Type Culture Collection (Manassas, VA, USA). All reagents related to animal cell culture were purchased from Life Technologies (Big Cabin, OK, USA). Cells were cultured in Dulbecco’s modified Eagle’s medium. All media contained 10% fetal bovine serum, 100 units/ml penicillin, and 0.1 mg/ml streptomycin. Cells were cultured at 37°C in a 5%-CO_2_ humidified environment. Cells (1×10^4^ cells/well) were plated on 12-well plates and counted every 24 hours for 5 days using the ADAM-MC automatic cell counter (NanoEnTek Inc., Seoul, South Korea).

Total RNA was isolated from cultured cells using TRIzol (Invitrogen, Carlsbad, CA, USA) according to the manufacturer’s instructions. For quantitative real-time reverse transcription-polymerase chain reaction (RT-PCR), cDNAs were synthesized from 4 μg of total RNA using random hexamer primers and SuperScript reverse transcriptase II (Invitrogen) following the manufacturer’s instructions (Supplementary Table 3). Diluted cDNAs were analyzed for qPCR using the SYBR Green PCR Master Mix (Applied Biosystems, Carlsbad, CA, USA) and gene-specific primers, and then subjected to RT-PCR quantification using the ABI PRISM 7300 RT-PCR System (Applied Biosystems).

For the stable overexpression of AR, the fragment encoding the full-length cDNA of AR was cloned into the pLL-CMV-puro lentiviral vector. Plasmid DNAs and a lentiviral packaging mix containing an envelope and packaging vector were transfected into human embryonic kidney (HEK293T) cells according to the manufacturer’s instructions to produce lentiviruses packed with AR cDNA cassettes. Positive cells harboring AR cDNA cassette were selected by 1 μg/ml puromycin (Sigma-Aldrich, St. Louis, MO, USA) selection after infection. For the knockdown assay, targeting small interfering RNA (siRNA) and non-targeting control siRNA were transfected into cells utilizing Lipofectamine RNAiMax reagent (Invitrogen) following the manufacturer’s protocols. The sequences of targeting oligo duplex against FOXA1 were as follows: 5`–GAGAGAAAAAATCAACAGCTT–3`(sense) and 5`–GCTGTTGATTTTTTCTCTCTT–3`(antisense) (Integrated DNA Technologies Inc., Coralville, IA, USA).

Cell viability was determined by EZ-Cytox Cell Viability Assay Kit (Daeil Lab Service, Seoul, South Korea) based on the cleavage of the tetrazolium salt to water-soluble formazan by succinate-tetrazolium reductase. Cells in suspension with siRNA mixtures were transferred to 96-well plate (5x10^3^ cells/well) followed by medium changed the next day. After 48 hours, Ez-Cytox reagent (10 ul/well) was added and absorbance (OD_450_) was detected at 450 nm after 4 hours.

For Western blot analysis, cultured cells were washed twice with ice-cold phosphate-buffered saline and harvested in whole-cell lysis buffer (1% sodium dodecyl sulfate, 60 mM Tris-HCl, pH 6.8). Protein concentrations were measured by the bicinchoninic acid assay. Equal amounts of protein extracts were subjected to sodium dodecyl sulfate–polyacrylamide gel electrophoresis (SDS–PAGE) and transferred onto nitrocellulose transfer membranes (Whatman GmbH, Dassel, Germany). The membranes were blocked in 5% (w/v) non-fat Difco™ skimmed milk (BD Biosciences, San Jose, CA, USA), followed by incubation with the primary antibodies in 1% bovine serum albumin. The following antibodies were used: anti-AR (custom-made), anti-ERα (Santa Cruz Biotechnology Inc., Santa Cruz, CA, USA), α-tubulin (Calbiochem, Brookfield, WI, USA), and FOXA1 (Cell Signaling Technology, Danvers, MA, USA).

Luciferase activities in whole cell lysates were measured using the Dual-Luciferase Reporter Assay System^®^ (Promega Corp., Madison, WI, USA). Using a pRL-SV40 construct (Promega Corp.), luciferase activity was normalized to each cell lysate’s *Renilla* luciferase activity levels.

### TMA study

A previous study cohort was selected to investigate the clinical implications of immunohistochemically determined AR and FOXA1 expression levels on breast cancer patients’ survival outcomes. Immunohistochemical AR expression was evaluated from TMA blocks of 931 patients treated between November 1999 and August 2005 [28]. Using consecutive slides of prior TMA blocks, FOXA1 expression was evaluated by IHC and was determined to be uninterpretable in 65 cases. The remaining patients (n = 866) who had both readable AR and FOXA1 expression were analyzed in the present study.

Patient demographics, histopathology of primary tumor, treatment patterns and survival rates were retrospectively obtained from medical records. Tumor-node-metastasis (TNM) stage was determined from the 6th American Joint Committee on Cancer criteria. Histological grade was assessed by the modified Bloom-Richardson classification. DFS time was measured from the date of the first curative surgery to the date of the first local, regional, or distant recurrence or death without any type of relapse. OS time was measured from the date of the first operation to the date of the last follow-up or death from any cause.

Immunohistochemical staining was performed using prior formalin-fixed, paraffin-embedded TMA tumor blocks as detailed in procedure descriptions from a previous study [28]. Using consecutive TMA sections, FOXA1 expression was evaluated and primary antibody against FOXA1 (2F83, 1:4,000; Abcam, Cambridge, United Kingdom) was used. AR, ER, PR, HER2 and Ki-67 expression were obtained from previous results. Tumors with ≥ 10% positively nuclear-stained cells were considered positive for AR expression (Figures 3A and 3C). Considering the proportion and staining intensity, FOXA1 expression was categorized as 0 (negative), 1 (weak), 2 (moderate), and 3 (strong). An arbitrary cutoff point of ≥ 2 was applied to determine FOXA1-positivity (Figures 3B and 3D). Tumors with ≥ 1% nuclear-stained cells were considered positive for ER and PR based on the American Society of Clinical Oncology/College of American Pathologists (ASCO/CAP) guidelines [36]. HER2 status was evaluated using the HercepTest™ (DAKO) and was interpreted as 0, 1+, 2+, or 3+ according to the ASCO/CAP guidelines [37]. In cases with HER2 2+ results, fluorescence in situ hybridization (FISH) was performed using a PathVysion HER2 DNA Probe Kit (Vysis, Downers Grove, IL, USA). HER2 gene amplification was classified as a case with either HER2 gene/chromosome 17 copy number ratio ≥ 2.0 or < 2.0, along with an average HER2 copy number ≥ 6.0 signals/cell as determined by ASCO/CAP guidelines [37]. HER2 was considered positive in cases with a 3+ IHC score or gene amplification by FISH regardless of the HER2 IHC result. Ki-67 levels were scored by counting the number of positively stained nuclei and were expressed as a percentage of total tumor cells. Based on the IHC scores or FISH findings of ER, PR, HER2, and Ki-67 expression, breast cancer subtypes were categorized as follows: luminal A (ER+ and/or PR+, HER2–, and Ki-67 < 15%); luminal B (ER+ and/or PR+, HER2–, and Ki-67 ≥ 15% or ER+ and/or PR+ and HER2+ irrespective of Ki-67 expression); HER2-positive (ER–, PR–, and HER2+); and TNBC (ER–, PR–, and HER2–).

This study was approved by the Institutional Review Board of Severance Hospital, Yonsei University Health System, Seoul, Republic of Korea (IRB No. 4-2016-0993). Written informed consent was waived and patient information was anonymized and deidentified prior to analysis.

### Statistical analysis

Web-based bioinformatics statistics including mutual exclusivity, correlation coefficient (*r*), HR with 95% CI, and a log-rank *p*-value were automatically calculated in a website and the results were displayed. An independent, two-sample *t*-test was used to compare the means of continuous numerical datasets. Differences between the groups were evaluated by a chi-square test. In order to analyze the downloaded METABRIC dataset and the TMA study, survival curves were plotted using the Kaplan-Meier method and group differences in survival time were investigated by a log-rank test. A Cox’s proportional hazards model was used to identify the variables that were independently associated with survival. All statistical tests were two-tailed and a *p*-value < 0.05 was considered statistically significant. SPSS version 23.0 (IBM Inc., Armonk, NY, USA) was used for all statistical analyses.

## SUPPLEMENTARY MATERIALS FIGURE AND TABLE


