# Supplementary material for: Lack of both androgen receptor and forkhead box A1 (FOXA1) expression is a poor prognostic factor in estrogen receptor-positive breast cancers
Source: Oncotarget. 2017 Sep 15;8(47):82940–55. doi: 10.18632/oncotarget.20937 (PMC5669940; doi:10.18632/oncotarget.20937)
Supplement: Supplementary file 1 [file oncotarget-08-82940-s001.pdf]

# Lack of both androgen receptor and forkhead box A1 (FOXA1) expression is a poor prognostic factor in estrogen receptor-positive breast cancers

## SUPPLEMENTARY MATERIALS

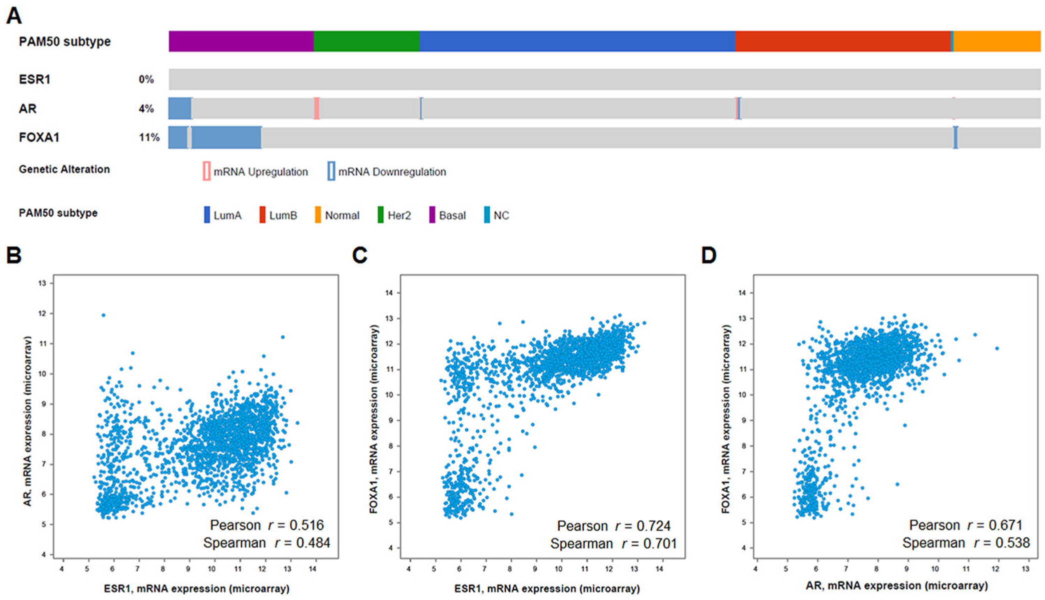


**Supplementary Figure 1: Genomic expression on the cBioPortal website.** **(A)** Genomic alteration is presented by the OncoPrint using the METABRIC dataset. Associations of mRNA expression are shown with correlation coefficients **(B)** between ESR1 and AR, **(C)** between ESR1 and FOXA1, and **(D)** between AR and FOXA1.

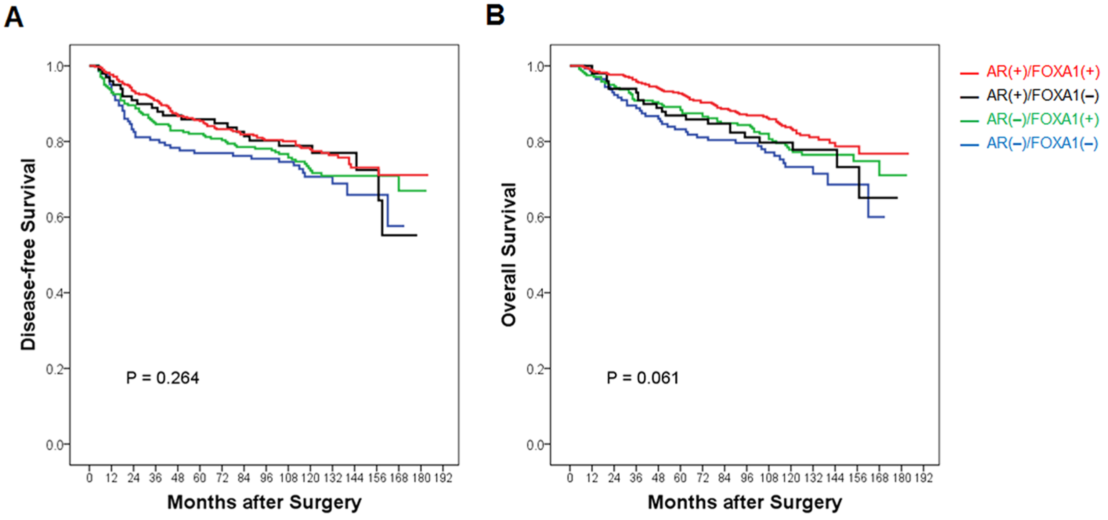


**Supplementary Figure 2: Survival curves according to AR and FOXA1 status in all patients of the TMA study.** **(A)** Disease-free survival and **(B)** Overall survival curves are shown according to immunohistochemical AR and FOXA1 status.

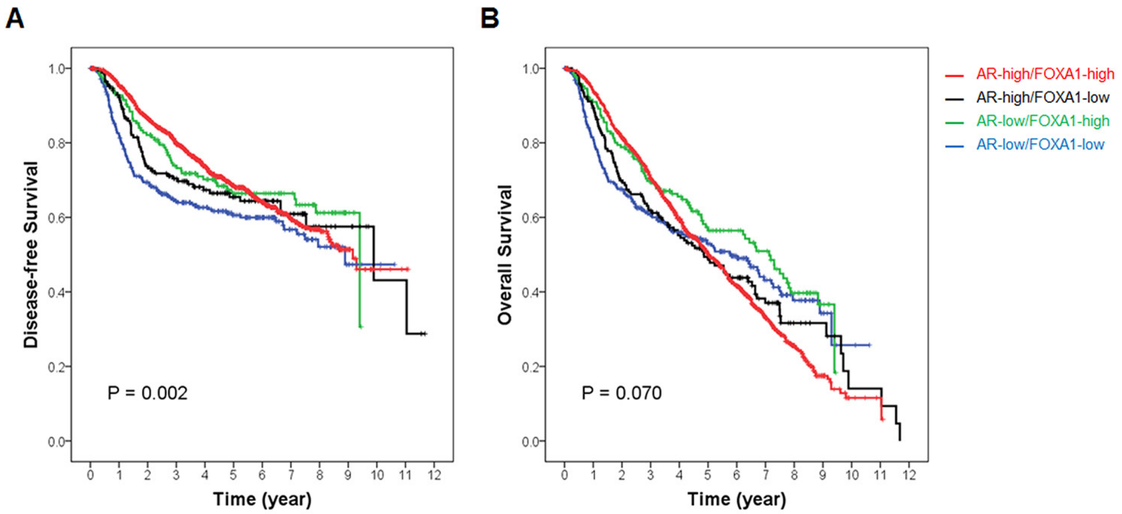


**Supplementary Figure 3: Survival curves according to AR and FOXA1 status in all patients of the METABRIC dataset.** **(A)** Disease-free survival and **(B)** Overall survival curves are presented according to AR and FOXA1 mRNA levels of the METABRIC dataset from the cBioPortal for Cancer Genomics (http://www.cbioportal.org).

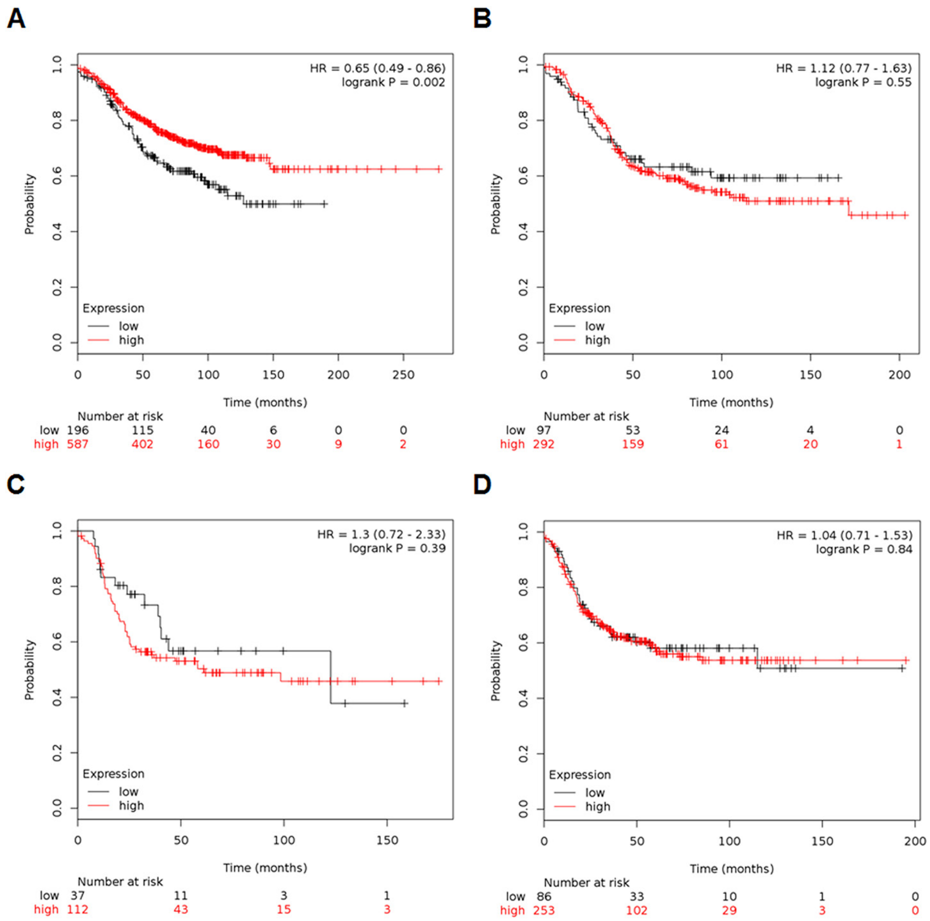


**Supplementary Figure 4: Relapse-free survival curves stratified by the intrinsic subtype in the KM Plotter.** Plots are generated according to AR and FOXA1 levels in each **(A)** luminal A, **(B)** luminal B, **(C)** HER2-enriched, and **(D)** basal subtype from the KM Plotter.

**Supplementary Table 1: Clinicopathological characteristics according to AR and FOXA1 expression in the whole population of the TMA study**

| Factor | AR(+)/ FOXA1(+) (n = 384, %) | AR(+)/ FOXA1(-) (n = 99, %) | AR(-)/ FOXA1(+) (n = 240, %) | AR(-)/ FOXA1(-) (n = 143, %) | *p*-value |
|---|---|---|---|---|---|
| Age (yrs) | | | | | |
| ≤ 50 | 241 (62.8) | 53 (53.5) | 162 (67.5) | 91 (63.6) | 0.115 |
| > 50 | 143 (37.2) | 46 (46.5) | 78 (32.5) | 52 (36.4) | |
| Type | | | | | |
| Ductal | 347 (90.4) | 93 (93.9) | 207 (86.2) | 129 (90.2) | 0.158 |
| Lobular/ special | 37 (9.6) | 6 (6.1) | 33 (13.8) | 14 (9.8) | |
| Tumor stage | | | | | |
| pT1 | 207 (53.9) | 41 (41.4) | 88 (36.7) | 50 (35.0) | < 0.001 |
| pT2-4 | 177 (46.1) | 58 (58.6) | 152 (63.3) | 93 (65.0) | |
| Node stage | | | | | |
| pN0 | 204 (53.1) | 46 (46.5) | 120 (50.0) | 66 (46.2) | 0.424 |
| pN1-3 | 180 (46.9) | 53 (53.5) | 120 (50.0) | 77 (53.8) | |
| Histologic grade | | | | | |
| I/II | 325 (84.6) | 77 (77.8) | 145 (60.4) | 82 (57.3) | < 0.001 |
| III | 59 (15.4) | 22 (22.2) | 95 (39.6) | 61 (42.7) | |
| ER | | | | | |
| Positive | 356 (92.7) | 68 (68.7) | 141 (58.8) | 60 (42.0) | < 0.001 |
| Negative | 28 (7.3) | 31 (31.3) | 99 (41.2) | 83 (58.0) | |
| PR | | | | | |
| Positive | 307 (79.9) | 61 (61.6) | 122 (50.8) | 49 (34.3) | < 0.001 |
| Negative | 77 (20.1) | 38 (38.4) | 118 (49.2) | 94 (65.7) | |
| HER2 | | | | | |
| Negative | 293 (76.3) | 64 (64.6) | 180 (75.0) | 117 (81.8) | 0.023 |
| Positive | 91 (23.7) | 35 (35.4) | 60 (25.0) | 26 (18.2) | |
| Ki-67 (n = 864) | | | | | |
| < 15% | 345 (90.1) | 89 (90.8) | 156 (65.0) | 73 (51.0) | < 0.001 |
| ≥ 15% | 38 (9.9) | 9 (9.2) | 84 (35.0) | 70 (49.0) | |
| Breast cancer subtypes (n = 864) | | | | | |
| Luminal A | 267 (69.7) | 52 (53.1) | 98 (40.8) | 42 (29.4) | < 0.001 |
| Luminal B | 89 (23.2) | 17 (17.3) | 53 (22.1) | 26 (18.2) | |
| HER2-positive | 18 (4.7) | 20 (20.4) | 21 (8.8) | 16 (11.2) | |
| TNBC | 9 (2.3) | 9 (9.2) | 68 (28.3) | 59 (41.3) | |
| Type of surgery | | | | | |
| BCS | 116 (30.2) | 28 (28.3) | 68 (28.3) | 37 (25.9) | 0.800 |
| TM | 268 (69.8) | 71 (71.7) | 172 (71.7) | 106 (74.1) | |
| Radiation therapy | | | | | |
| Not done | 201 (52.3) | 49 (49.5) | 125 (52.1) | 75 (52.4) | 0.964 |
| Done | 183 (47.7) | 50 (50.5) | 115 (47.9) | 68 (47.6) | |
| Chemotherapy | | | | | |
| Not done | 58 (15.1) | 16 (16.2) | 25 (10.4) | 15 (10.5) | 0.210 |
| Done | 326 (84.9) | 83 (83.8) | 215 (89.6) | 128 (89.5) | |
| Endocrine therapy | | | | | |
| Not done | 58 (15.1) | 37 (37.4) | 110 (45.8) | 87 (60.8) | < 0.001 |
| Done | 326 (84.9) | 62 (62.6) | 130 (54.2) | 56 (39.2) | |

AR: androgen receptor, ER: estrogen receptor, PR: progesterone receptor, HER2: human epidermal growth factor receptor 2, TNBC: triple-negative breast cancer, BCS: breast conservation surgery, TM: total mastectomy.

**Supplementary Table 2: Clinicopathological characteristics according to AR and FOXA1 mRNA status in the whole population of the METABRIC dataset**

| Factor | AR-high/ FOXA1-high (n, %) | AR-high/ FOXA1-low (n, %) | AR-low/ FOXA1-high (n, %) | AR-low/ FOXA1-low (n, %) | *p*-value |
|---|---|---|---|---|---|
| Age (yrs) | | | | | |
| ≤ 50 | 223 (17.1) | 38 (22.9) | 40 (23.8) | 119 (37.5) | < 0.001 |
| > 50 | 1,080 (82.9) | 131 (77.1) | 128 (76.2) | 198 (62.5) | |
| TNM stage | | | | | |
| Stage I | 403 (31.1) | 45 (26.9) | 67 (40.1) | 84 (27.0) | 0.004 |
| Stage II | 757 (58.5) | 92 (55.1) | 87 (52.1) | 186 (59.8) | |
| Stage III | 134 (10.4) | 30 (18.0) | 13 (7.8) | 41 (13.2) | |
| Histologic grade | | | | | |
| I/II | 748 (60.1) | 49 (30.2) | 84 (50.9) | 52 (16.8) | < 0.001 |
| III | 496 (39.9) | 113 (69.8) | 81 (49.1) | 257 (83.2) | |
| ER* | | | | | |
| Positive | 1,168 (91.1) | 97 (58.1) | 148 (89.2) | 72 (23.4) | < 0.001 |
| Negative | 114 (8.9) | 70 (41.9) | 18 (10.8) | 236 (76.6) | |
| PR† | | | | | |
| Positive | 865 (66.4) | 54 (31.8) | 86 (51.2) | 26 (8.2) | < 0.001 |
| Negative | 438 (33.6) | 116 (68.2) | 82 (48.8) | 291 (91.8) | |
| HER2† | | | | | |
| Negative | 1,148 (88.1) | 125 (73.5) | 146 (86.9) | 297 (93.7) | < 0.001 |
| Positive | 155 (11.9) | 45 (26.5) | 22 (13.1) | 20 (6.3) | |
| PAM50 subtype | | | | | |
| Luminal A | 632 (48.7) | 19 (11.3) | 60 (35.7) | 4 (1.3) | < 0.001 |
| Luminal B | 388 (29.9) | 20 (11.9) | 66 (39.3) | 6 (1.9) | |
| HER2 | 146 (11.2) | 45 (26.8) | 25 (14.9) | 22 (6.9) | |
| Basal | 23 (1.8) | 45 (26.8) | 7 (4.2) | 250 (78.9) | |
| Normal | 110 (8.5) | 39 (23.2) | 10 (6.0) | 35 (11.0) | |
| Type of surgery | | | | | |
| BCS | 499 (38.6) | 63 (38.2) | 74 (44.3) | 143 (46.3) | 0.054 |
| TM | 795 (61.4) | 102 (61.8) | 93 (55.7) | 166 (53.7) | |
| Radiotherapy | | | | | |
| Not done | 576 (44.2) | 53 (31.2) | 68 (40.5) | 96 (30.3) | < 0.001 |
| Done | 727 (55.8) | 117 (68.8) | 100 (59.5) | 221 (69.7) | |
| Chemotherapy | | | | | |
| Not done | 1,134 (87.0) | 120 (70.6) | 140 (83.3) | 155 (48.9) | < 0.001 |
| Done | 169 (13.0) | 50 (29.4) | 28 (16.7) | 162 (51.1) | |
| Hormone therapy | | | | | |
| Not done | 409 (31.4) | 80 (47.1) | 53 (31.5) | 208 (65.6) | < 0.001 |
| Done | 894 (68.6) | 90 (52.9) | 115 (68.5) | 109 (34.4) | |

*Positive criteria of ER was measured by immunohistochemistry in the METABRIC dataset.

†Positive criteria of PR and HER2 were defined as expression status in the METABRIC dataset.

**Supplementary Table 3: Primers used for real-time reverse transcription-polymerase chain reaction (RT-PCR)**

| Gene symbol | GenBank Accession No. | Sequence |
|---|---|---|
| ESR1 | NM_000125 | FW: ATGACTATGCTTCAGGCTACCATT<br>RV: GTGGCTGGACACATATAGTCGTTA |
| AR | NM_000044 | FW: CGACCAGATGGCTGTCATTC<br>RV: TGTGCATGCGGTACTCATTG |
| FOXA1 | NM_004496 | FW: ACTCCTTCAACCACCCGTTC<br>RV: GCGAGTATTGCAGTGCCTGT |
